# Supplementary figures and images for: BMP signaling maintains auricular chondrocyte identity and prevents microtia development by inhibiting protein kinase A
Source: eLife. 2024 May 1;12:RP91883. doi: 10.7554/eLife.91883 (PMC11062634; doi:10.7554/eLife.91883)

Figure 5D

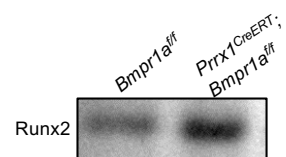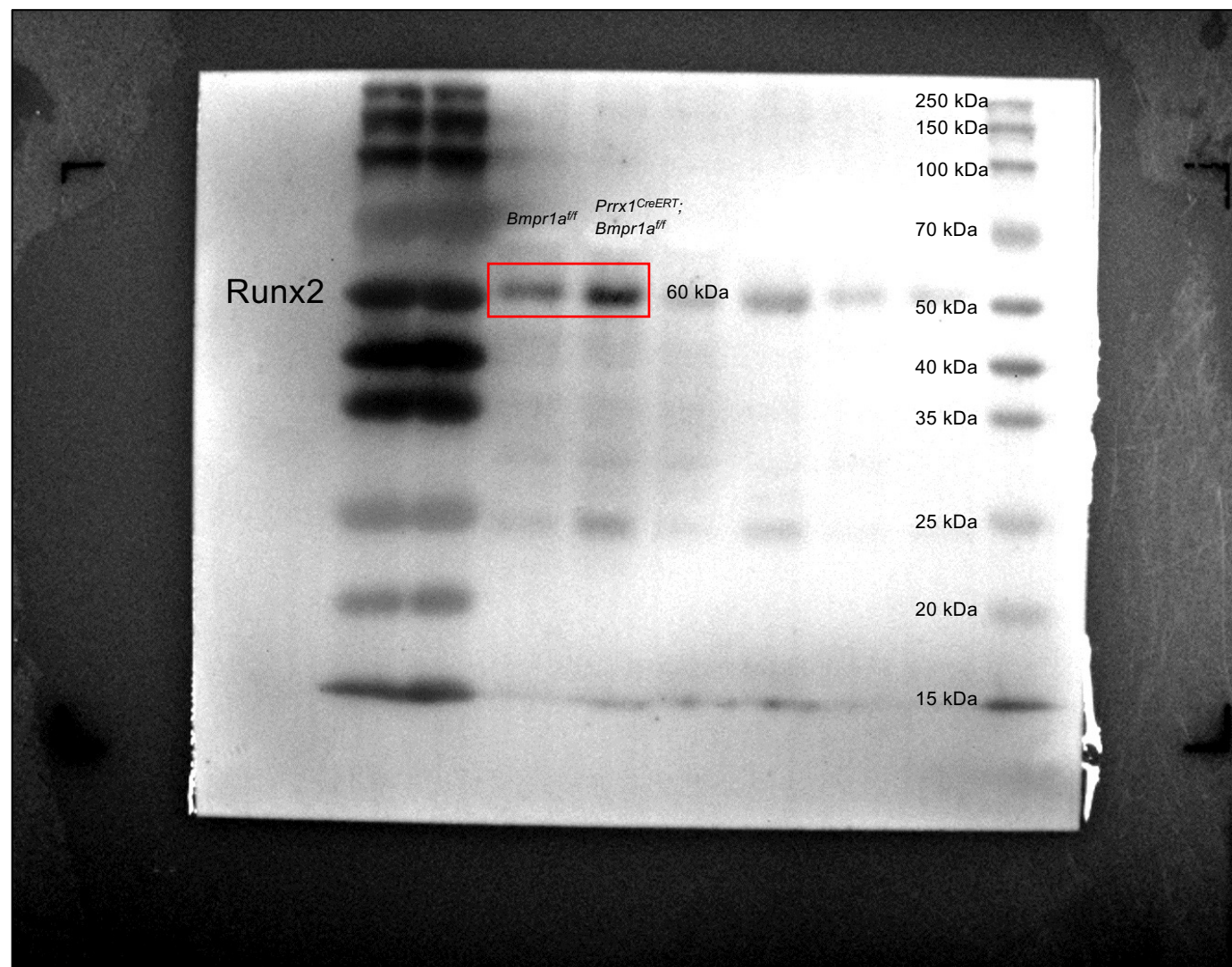

Figure 5D

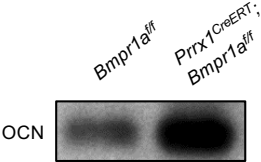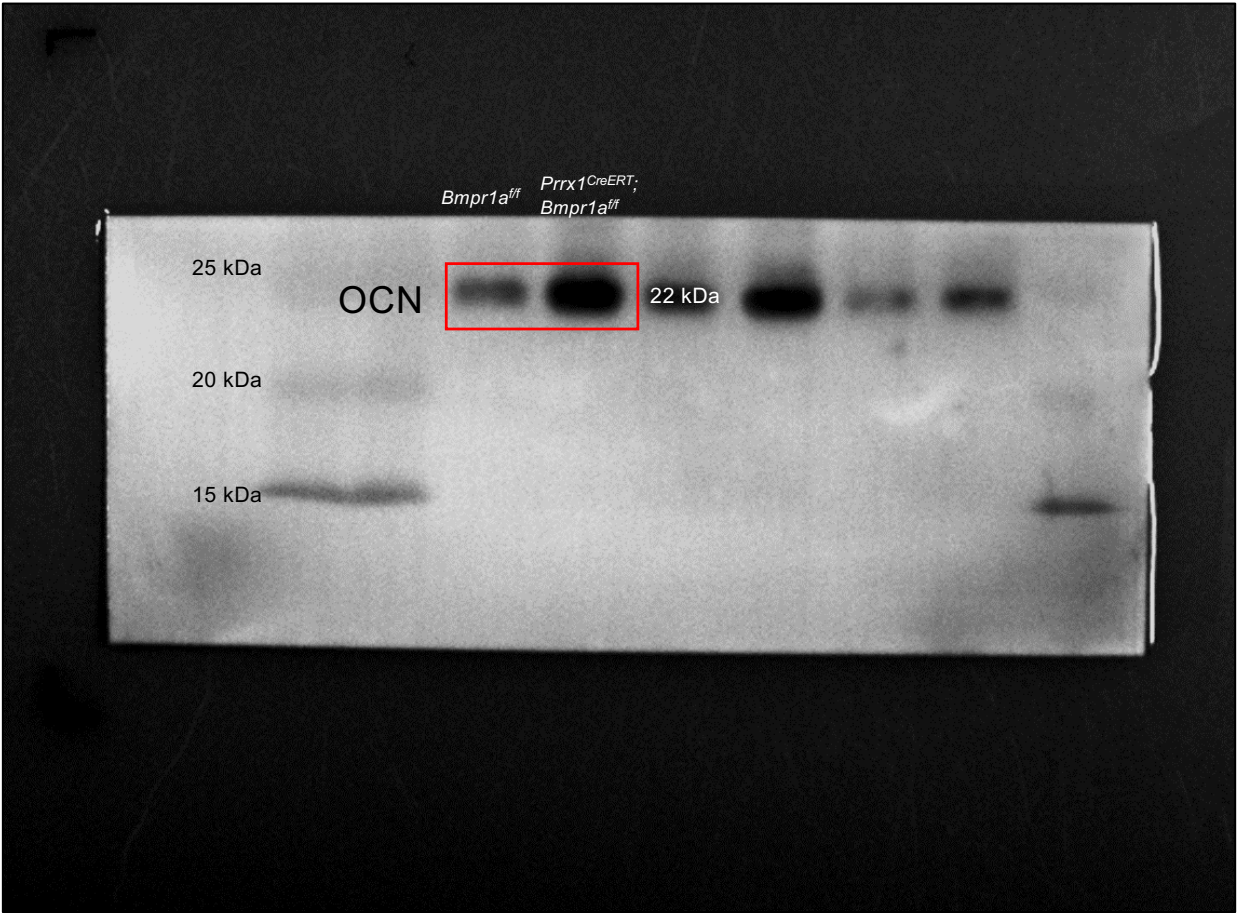

Figure 5D

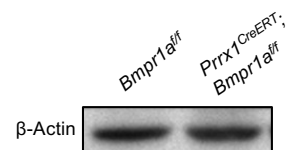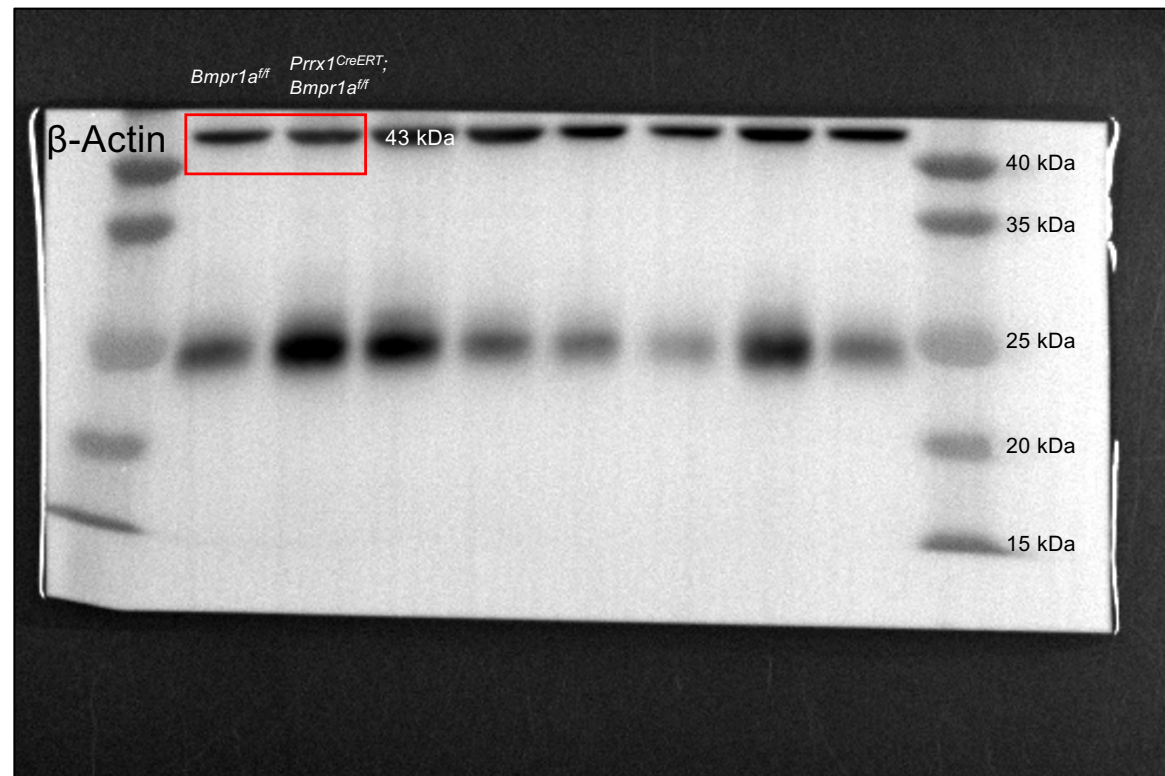

Supplement: Figure 5—source data 1. [file elife-91883-fig5-data1.pdf]

Figure 6B

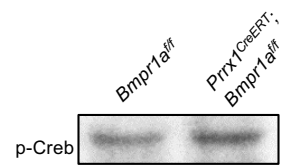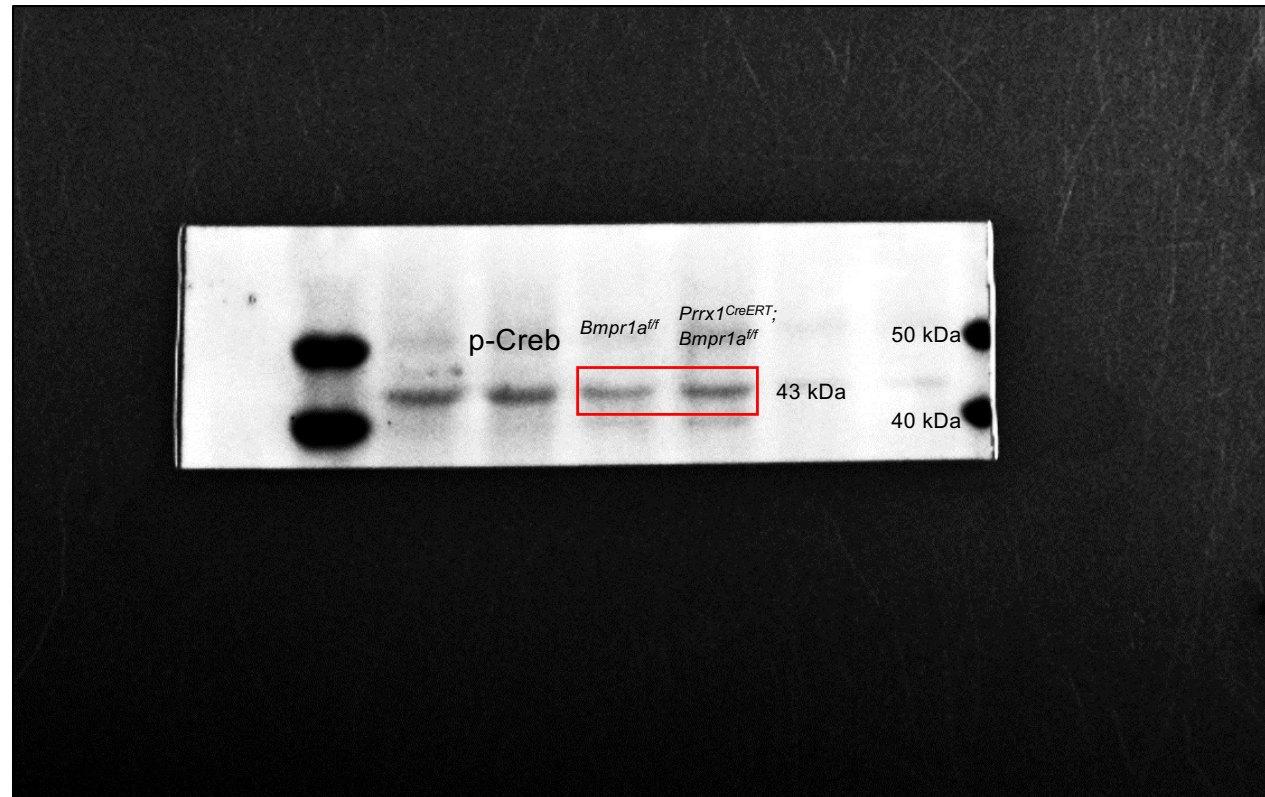

Figure 6B

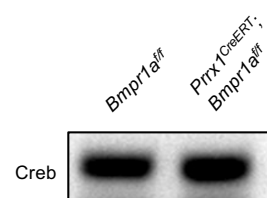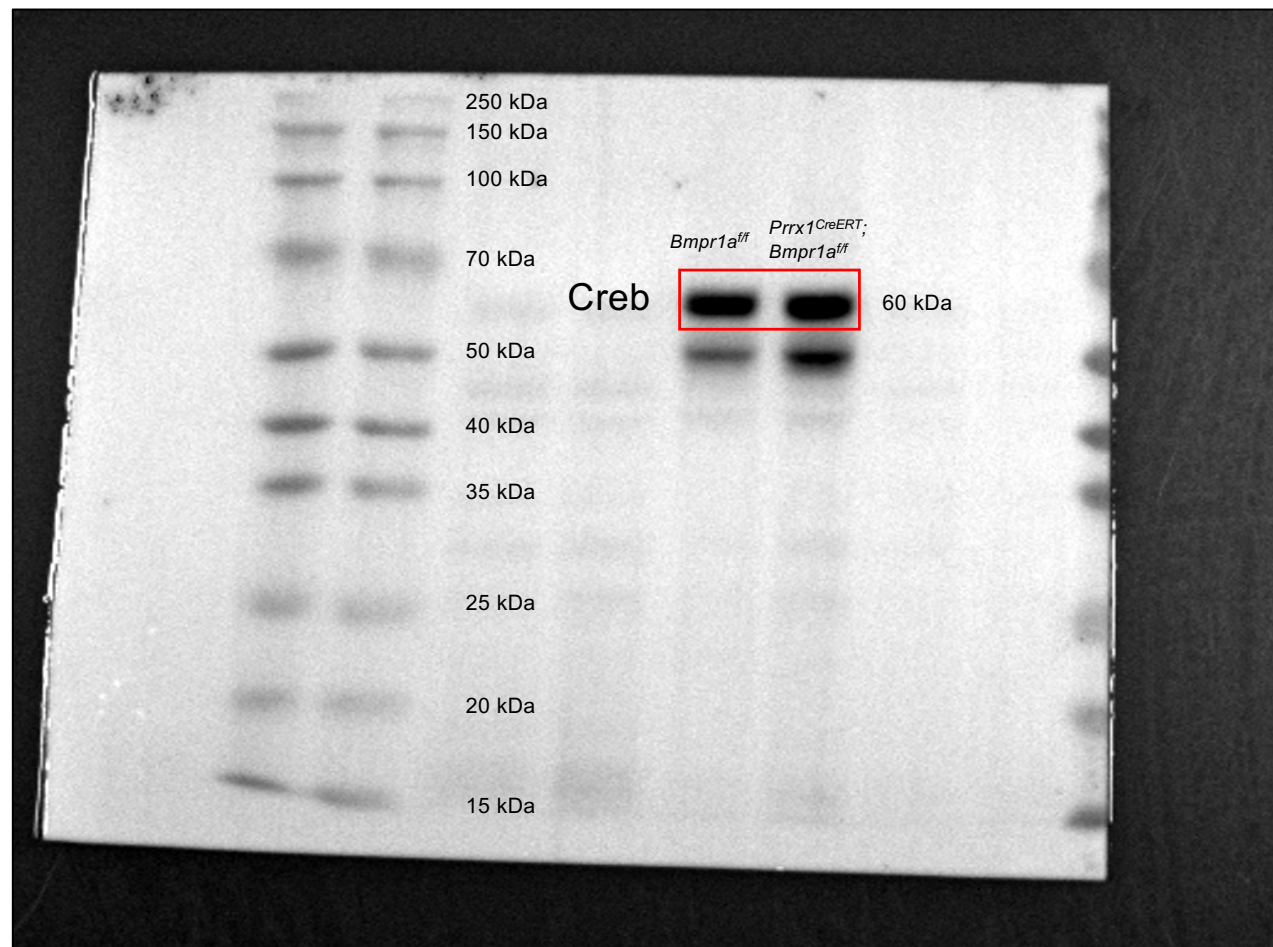

Figure 6B

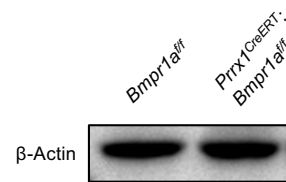

$\beta$ -Actin

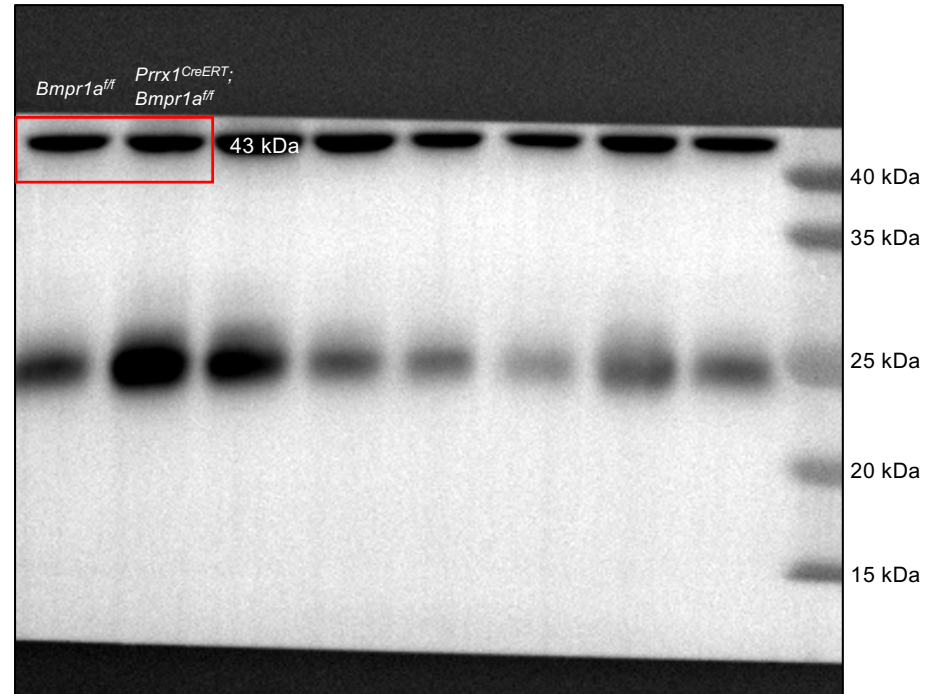

Supplement: Figure 6—source data 1. [file elife-91883-fig6-data1.pdf]

Figure 7G

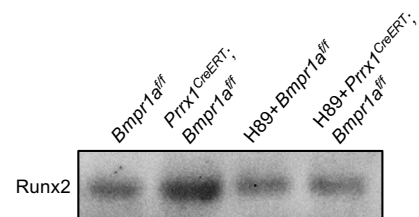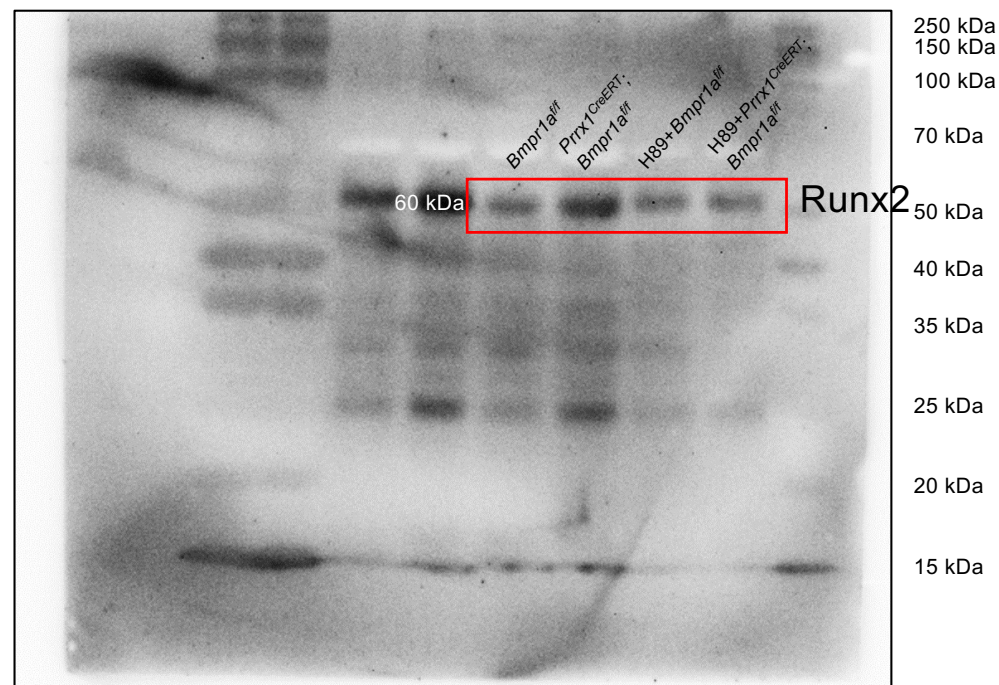

Figure 7G

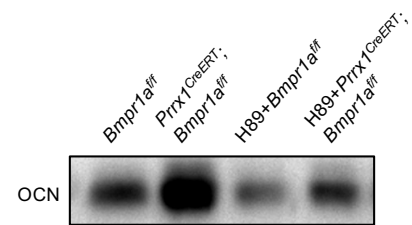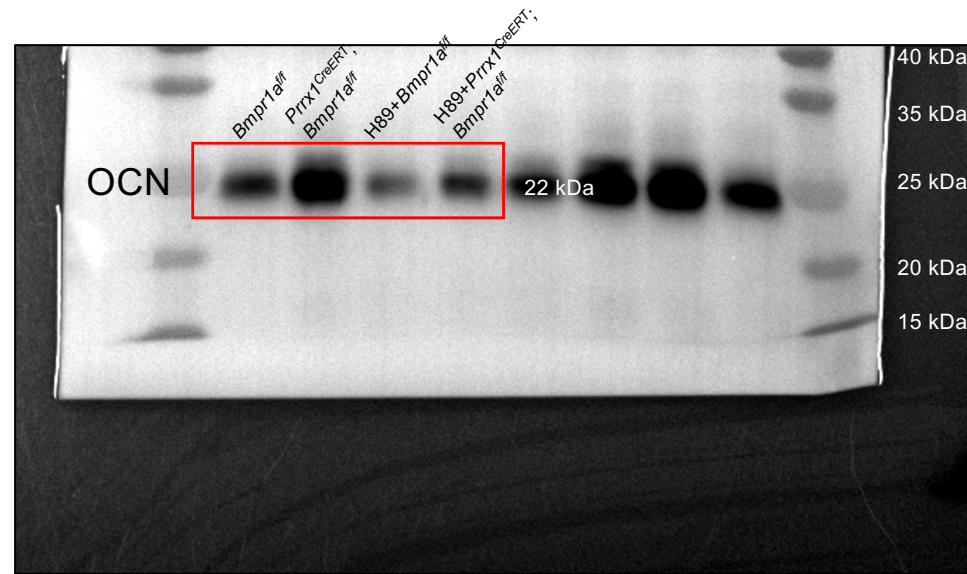

Figure 7G

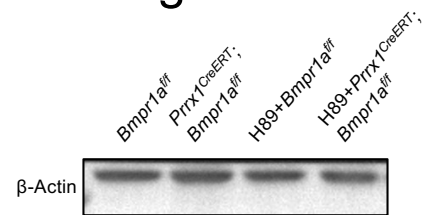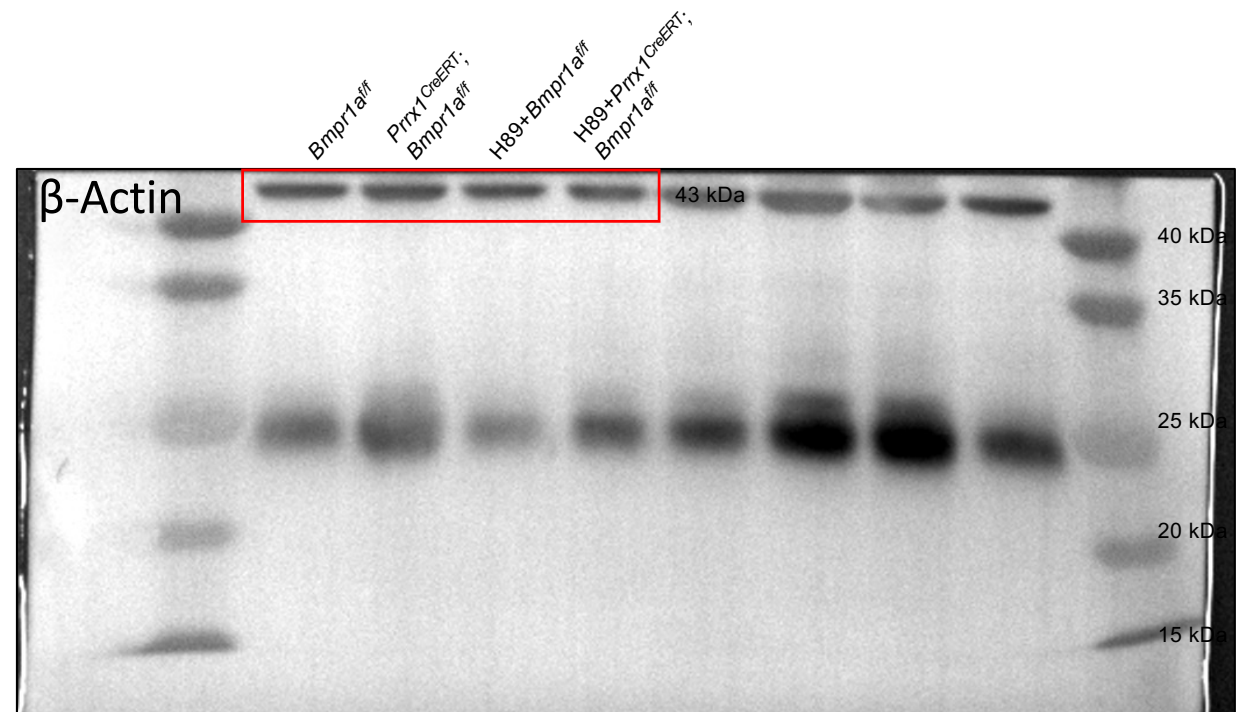

Supplement: Figure 7—source data 1. [file elife-91883-fig7-data1.pdf]
